# A232 TEMPORAL TRENDS IN RE-HOSPITALIZATION RATES AMONG PATIENTS WITH INFLAMMATORY BOWEL DISEASE IN THE POST-BIOLOGIC ERA

**DOI:** 10.1093/jcag/gwad061.232

**Published:** 2024-02-14

**Authors:** C Dziegielewski, M Pugliese, J Begum, E I Benchimol, J McCurdy, S Murthy

**Affiliations:** Gastroenterology, University of Ottawa, Ottawa, ON, Canada; Ottawa Hospital Research Institute, Ottawa, ON, Canada; ICES uOttawa, Ottawa, ON, Canada; University of Toronto, Toronto, ON, Canada; Gastroenterology, University of Ottawa, Ottawa, ON, Canada; Gastroenterology, University of Ottawa, Ottawa, ON, Canada

## Abstract

**Background:**

Hospitalization for severe inflammatory bowel disease (IBD) flares or complications are a major source of morbidity and healthcare expenditure.

**Aims:**

To assess temporal trends in 30-day and 90-day re-hospitalization rates among persons with Crohn’s disease (CD) and ulcerative colitis (UC) in the post-biologic era.

**Methods:**

We conducted a population-based study of all non-elective IBD-related hospitalizations among patients with CD and UC in Ontario, Canada between April 1, 2002 and March 31, 2020. We identified individuals, admissions, and variables of interest from Ontario population health administrative datasets housed at IC/ES. We performed multivariable logistic regression analysis to evaluate the association between time period of hospitalization (2002-2007 vs. 2002-2007 vs. 2007-2012 for CD; 2004-2008 vs. 2008-2012 vs. 2012-2016 vs. 2016-2020 for UC) and rates of 30-day and 90-day re-hospitalization, adjusting for patient age, sex, co-morbidities, residential setting, income quintile, hospital type of initial admission, and clustering of hospital admissions within patients. We excluded patients with elective admissions or length of stay ampersand:003C24 hours, and those without continuous valid Ontario health care registration during the 90-day period following hospital discharge.

**Results:**

There were 18,625 hospitalizations among 14,868 patients with CD, and 10,830 hospitalizations among 9,264 patients with UC. The 30-day re-hospitalization rate was 8.5% for patients with CD and 9.7% for patients with UC, while the respective 90-day re-hospitalization rates were 15.0% and 13.8%. For CD, re-admission rates differed across the 3 time periods (pampersand:003C0.0001 for 30-day and p=0.012 for 90-day). For UC, re-hospitalization rates differed across the 4 time periods for 30-day re-hospitalization (p=0.048), but not 90-day (p=0.080). There was a higher relative odds of CD-related re-hospitalization in 2002-2007 as compared to 2012-2017 (adjusted odds ratio [aOR] 1.32, 95% confidence interval [CI] 1.16-1.50 for 30-day; aOR 1.14, 95% CI [1.03-1.26] for 90-day). For UC, there was a higher relative odds of re-hospitalization in 2012-2016 as compared to 2016-2020 (aOR 1.26, 95% CI [1.05-1.52] for 30-day; aOR 1.22, 95% CI [1.04-1.43] for 90-day).

**Conclusions:**

Up to 15% of patients with IBD in Ontario are re-admitted to hospital within 90 days of hospital discharge. The risk of re-hospitalization may have decreased over time in the post-biologic era. Our findings could be explained by changing access to inpatient and/or outpatient resources, improvements to medical and/or surgical care, shifting patient behaviour with respect to healthcare resource utilization, or residual confounding. These results require validation in other jurisdictions.

**Funding Agencies:**

None

